# Cu and Zn Interactions with Peptides Revealed by High-Resolution Mass Spectrometry

**DOI:** 10.3390/ph15091096

**Published:** 2022-08-31

**Authors:** Monica Iavorschi, Ancuța-Veronica Lupăescu, Laura Darie-Ion, Maria Indeykina, Gabriela Elena Hitruc, Brîndușa Alina Petre

**Affiliations:** 1 Department of Biomedical Sciences, Faculty of Medicine and Biological Sciences, Stefan cel Mare University of Suceava, 13 University, 720229 Suceava, Romania; 2 Faculty of Chemistry, Al. I. Cuza University of Iasi, 11 Carol I, 700506 Iasi, Romania; 3 Emanuel Institute for Biochemical Physics, Russian Academy of Sciences, 119334 Moscow, Russia; 4 Physical Chemistry of Polymers Department, Petru Poni Institute of Macromolecular Chemistry, 41A Gr. Ghica Voda Alley, 700487 Iasi, Romania; 5 Center for Fundamental Research and Experimental Development in Translation Medicine—TRANSCEND, Regional Institute of Oncology, 2-4 General Henri Mathias Berthelot, 700483 Iași, Romania

**Keywords:** neurological peptides, metal ion interactions, MALDI-MS, ESI-MS, AFM

## Abstract

Alzheimer’s disease (AD) is a progressive neurodegenerative disease characterized by abnormal extracellular amyloid-beta (Aβ) peptide depositions in the brain. Among amorphous aggregates, altered metal homeostasis is considered a common risk factor for neurodegeneration known to accelerate plaque formation. Recently, peptide-based drugs capable of inhibiting amyloid aggregation have achieved unprecedented scientific and pharmaceutical interest. In response to metal ions binding to Aβ peptide, metal chelation was also proposed as a therapy in AD. The present study analyzes the interactions formed between NAP octapeptide, derived from activity-dependent neuroprotective protein (ADNP), amyloid Aβ(9–16) fragment and divalent metal ions such as Cu and Zn. The binding affinity studies for Cu and Zn ions of synthetic NAP peptide and Aβ(9–16) fragment were investigated by matrix-assisted laser desorption/ionization mass spectrometry (MALDI-MS), electrospray ion trap mass spectrometry (ESI-MS) and atomic force microscopy (AFM). Both mass spectrometric methods confirmed the formation of metal–peptide complexes while the AFM technique provided morphological and topographic information regarding the influence of metal ions upon peptide crystallization. Our findings showed that due to a rich histidine center, the Aβ(9–16) fragment is capable of binding metal ions, thus becoming stiff and promoting aggregation of the entire amyloid peptide. Apart from this, the protective effect of the NAP peptide was found to rely on the ability of this octapeptide to generate both chelating properties with metals and interactions with Aβ peptide, thus stopping its folding process.

## 1. Introduction

Addition of metal ions to peptides or proteins normally involves chelation of the metal ion at several amino acids that expose Lewis-basic properties. Lewis bases are nucleophile species that donate an electron pair to an electrophile to form a chemical bond. Thus, in the case of peptides, the nucleophile sites are found both in the covalent amide linkages and the amino acid side chains. The chelation provided by the amide carbonyl oxygen, known as charge-solvation binding, is a characteristic of alkali metal ions and Ca^2+^ or Mg^2^^+^ ions. Alternatively, the deprotonation of the amide nitrogen motif is considered to be distinctive for more active metal ions such as Co^2^^+^, Ni^2^^+^, Cu^2^^+^, Zn^2^^+^, Pd^2^^+^and Cd^2^^+^ [1].

Matrix-assisted laser desorption/ionization mass spectrometry (MALDI-MS) is a powerful analytical tool for the analysis of various biomolecules and synthetic polymers. When studying biological systems with MALDI-MS, it is very important to know whether the non-covalently bound complexes that are stable under physiological conditions are also able to survive laser desorption and ionization processes, which occur inside the ionization source. The electrospray ionization mass spectrometry (ESI-MS) is a powerful tool that in recent years has provided considerable information on non-covalent interactions between proteins and ligands, including the stoichiometry and affinity of complexes. This “soft” ionization technique allows non-covalent complexes of proteins to be admitted to the gas phase for detection and investigation according to their “charge-state families” [2]. Furthermore, different research reports have shown that during the electrospray ionization mass spectrometry ESI-MS, the non-covalent protein–metal interactions in-solution are maintained during desolation and sample transfer to the gas phase [3]. Until now, a few studies were able to compare solution and gas phase chemistries of non-covalent and metal ion–biomolecule complexes using both, ESI and MALDI ionization sources [4,5,6].

Copper (Cu^+^ and Cu^2^^+^) and zinc (Zn^2^^+^) ions play important roles in many chemical and biochemical processes, such as oxidation, dioxygen transport and electron transfer [7,8,9]. Both metal ions have also been implicated in human neurodegenerative diseases, such as Alzheimer’s disease [10,11]. Proteins associated with neurodegenerative diseases interact with several transition metal ions, which leads to an aggregation process. Furthermore, redox-active metal ions such as iron and copper favor the occurrence of reactive oxygen species (ROS) promoted by Fenton reactions. This process leads to the formation of the highly reactive hydroxyl radical (OH^•^) responsible for stimulating oxidative stress in the cell by lipid membrane peroxidation, DNA damage and protein oxidation or misfolding [12].

Previous MALDI investigations using Aβ model peptides showed that peptide–copper ion complexes are formed by a reductive process which yields primarily [M + Cu(I)]^+^ ions [13], whereas the electrospray ionization method generates both [M + Cu(I)]^+^ and [M + Cu(II)-H]^+^ ions [14]. In principle, the formation of [M + Cu(I)]^+^ ions is provided by the basic amino acids such as arginine (Arg), lysine (Lys) and histidine (His) [13,15]. For NAP neuroprotective peptide and amyloidal fragment, the interaction between the Cu and the basic residues is described in terms of competitive binding [16,17]. However, few studies have been performed to determine Cu and Zn binding sites preference and their neurological analogues. So far, we have not found research literature reports on the competition between metal ions and different binding sites. Addressing these issues will elucidate (i) the chemistry of metal binding to the octapeptide NAP and the amyloid fragment Aβ(9–16); and (ii) the challenges of Cu and Zn ions as specific and competitive binding to a specific site in a biological mixture of two peptides. In this study we investigated the interactions formed between NAP neuroprotective peptide, Aβ(9–16) fragment and divalent metal ions such as Cu and Zn. Matrix-assisted laser desorption/ionization mass spectrometry (MALDI-MS), electrospray ion trap mass spectrometry (ESI-MS) and atomic force microscopy (AFM) were used to investigate the stoichiometries and affinity toward Cu and Zn ions of model synthetic NAP peptide and Aβ(9–16) fragment.

## 2. Results

### 2.1. Mass Spectrometric Analysis

Characterization of zinc and copper ions interaction with NAP neuroprotective peptide and Aβ(9–16) fragments was performed by ESI and MALDI-ToF MS in combination with collision-induced dissociation (CID) fragmentation experiments. As expected, in ESI-MS as well as MALDI-MS analysis, the obtained spectra highlighting the formation of metal–peptide complex. Also, peak assignments were achieved based on the theoretical expected ion values (*m*/*z*) and validated by isotopic pattern information.

**Figure 1:**
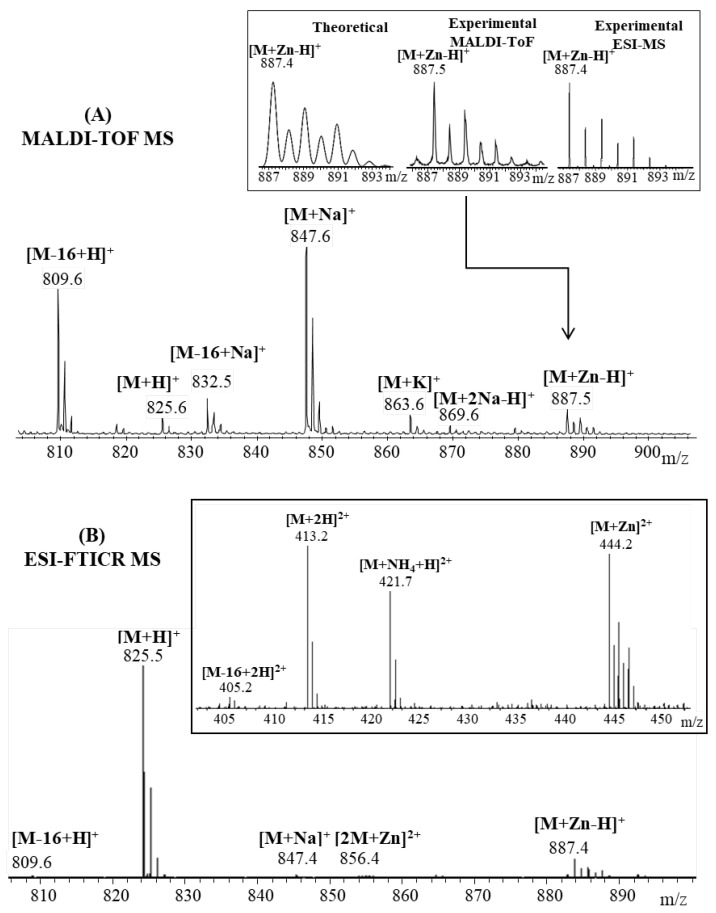
Enlarged region of mass spectra of NAP peptide incubated with Zn^2^^+^ ions acquired by (**A**) MALDI ToF (reflectron mode) with CHCA matrix and (**B**) ESI-FTICR, both in positive mode. Inserts: (**A**) details of theoretical and experimental isotopic patterns of [M + Zn-H]^+^ ion; (**B**) detail of the double-positive-charged peptide fragments.

Figure 1 presents the ESI and MALDI-ToF mass spectra of the octapeptide NAP incubated with Zn ions. Both mass spectra revealed the anticipated signal of metal–peptide complex, [M + Zn-H]^+^ at *m*/*z* 887.4. More precisely, [M + Zn−H]^+^ can be written as [M + H + Zn-2H]^+^ in order to account for the fact that the peptide loses two protons upon Zn^2+^complexation, and acquires an additional proton to form a singly charged species [18,19]. The most intense signals depicted in the MALDI spectrum were assigned to the sodium adduct ion ([M + Na]^+^, *m*/*z* 847.6) and deaminated [M-16 + H]^+^ ion (*m*/*z* 809.6), while the molecular ion [M + H]^+^ generated a small signal at *m*/*z* 825.6. The mechanism of deamination is photo chemically promoted by the laser light source and is favored by the C-terminus glutamine residue, where a rearrangement such as cyclization leads to pyroglutamic acid formation [20,21]. Additional signals observed in the mass spectrum at *m*/*z* 832.5, *m*/*z* 863.6 and *m*/*z* 869.6 correspond to different adducts with sodium and potassium ([M-16 + Na]^+^, [M + K]^+^, [M + 2Na-H]^+^). In contrast, ESI mass spectra generated a high abundant peak at *m*/*z* 825.5, which was attributed to the molecular ion [M + H]^+^. Furthermore, due to the use of a milder ionization technique such as ESI, only a small amount of peptide underwent a deamidation process and generated a small signal at *m*/*z* 809.6 [M-16 + H]^+^ ions. Signals assigned to the Zn^2+^–NAP metal complex were observed both in single- ([M + Zn−H]^+^ ion, *m*/*z* 887.4) and double-charged peptide fragments ([2M + Zn]^2^^+^ ion, *m*/*z* 856.4; [M + Zn]^2^^+^ ion, *m*/*z* 444.2). Additional doubly charged peptide ions generated during the ionization process were visualized at *m*/*z* 405.2, *m*/*z* 413.2 and *m*/*z* 421.7 and were assigned to the doubly protonated peptide ([M-16 + H]^+^, [M + H]^+^) and ammonium [M + NH_4_-H]^2^^+^ ion.

**Figure 2:**
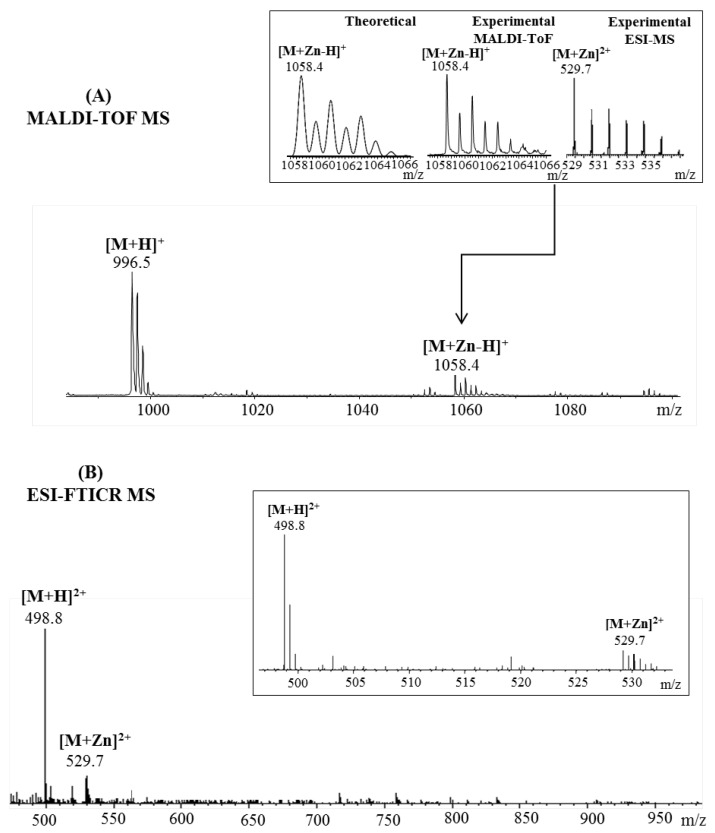
Enlarged region of mass spectra of Aβ(9–16) peptide incubated with Zn^2+^ ions acquired by (**A**) MALDI ToF (reflectron mode) with CHCA matrix and (**B**) ESI-FTICR, both in positive mode. Inserts: (**A**) details of theoretical and experimental isotopic patterns of [M + Zn-H]^+^and [M + Zn]^2^^+^ ions; (**B**) detail of the double-positive-charged peptide fragments.

In the case of Aβ(9–16) peptide, the MALDI ToF MS (Figure 2A) spectra contained a signal only for the single-charged peptide while the ESI MS spectra (Figure 2B) presented two peaks in the double-charged region. Thus, the zinc affinity toward the amyloidal peptide was confirmed by the presence of a small signal at *m*/*z* 1058.4 and *m*/*z* 529.7 corresponding to the zinc–peptide complex single- and double-charged species: [M + Zn-H]^+^ and [M + Zn]^2^^+^ ions. However, both ionization techniques showed an intense signal for the protonated 9–16 amyloid fragment that was observed at *m*/*z* 996.5 in the MALDI spectra and *m*/*z* 498.8 in the ESI experiment. The absence of single-charged species during electrospray ionization is due to the ability of histidine’s imidazole nitrogen atoms to act either as electron donors or acceptors in different cases [22] and thus, seize the second proton on its structure to form the doubly protonated ion [23].

**Figure 3:**
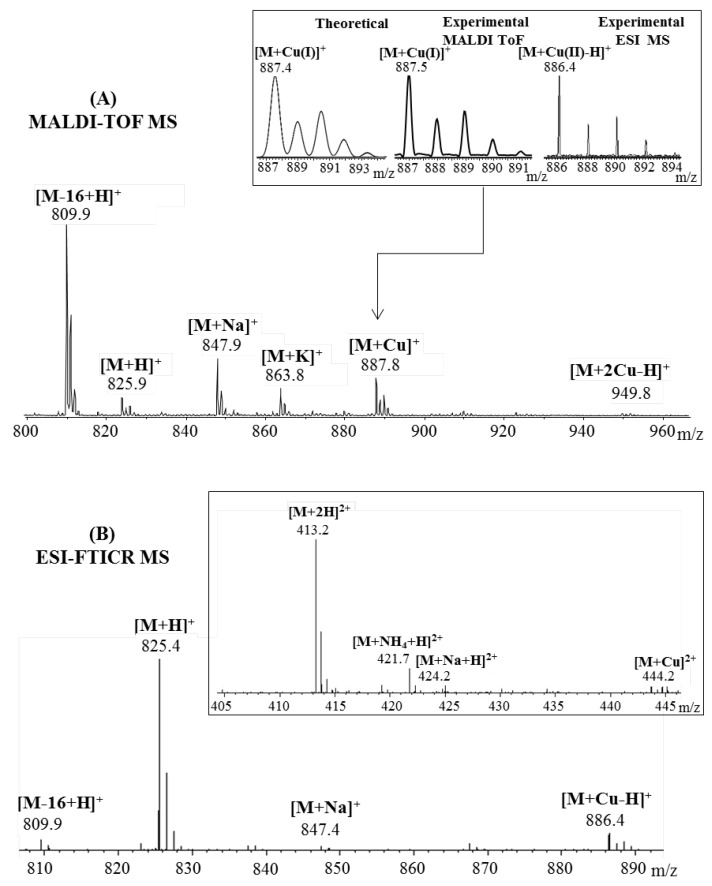
Enlarged region of mass spectra of NAP peptide incubated with Cu^2^^+^ ions acquired by (**A**) MALDI ToF (reflectron mode) with CHCA matrix and (**B**) ESI-FTICR, both in positive mode. Inserts: (**A**) details of theoretical and experimental isotopic patterns of [M + Cu-H]^+^ ion; (**B**) detail of the double-positive-charged peptide fragments.

Peptide interaction with copper (II) ions was also investigated by mass spectrometry. Figure 3 presents the MALDI and ESI MS spectra recorded after NAP octapeptide incubation with Cu^2^^+^ ions. At first glance, it can be easily observed that the peptides form stable complexes with metal ions. However, during matrix-assisted laser desorption ionization, species assigned to the copper (I)–peptide ions were observed at *m*/*z* 887.9 for the [M + Cu]^+^ species and *m*/*z* 949.8 for the [M + 2Cu-H]^2^^+^ containing two copper ions. The reduction of divalent metal ions is a process favored by both gas-phase charge exchange with matrix molecules and free electron capture [24]. Contrary to the MALDI spectra, where copper is present in reduced form almost exclusively, during ESI ionization, copper (II) ions maintain their charge state. Other signals observed in the mass spectra at *m*/*z* 825.9, *m*/*z* 847.9 and *m*/*z* 863.8 were assigned to the protonated peptide ([M + H]^+^) and adducts with sodium ([M + Na]^+^), potassium ([M + K]^+^). Furthermore, using ESI–MS, we found that NAP peptide was capable of binding only one copper ion. Thus, low-intensity signals attributed to the copper (II)–peptide complex were observed at *m*/*z* 886.4 corresponding to the single-charged [M + Cu-H]^+^ ions and *m*/*z* 444.2 assigned to the double-charged [M + Cu]^2+^ species. In addition to the peaks assigned to the metal ion–peptide complexes, the MALDI-MS spectra present an intense signal at *m*/*z* 809.9 characteristic of the deaminated peptide [M-16 + H]^+^, while the ESI experiment revealed two powerful peaks at *m*/*z* 825.4 and *m*/*z* 413.2 generated by the mono- and double-protonated peptide species. Additionally, during electrospray ionization, the peptide formed beside the single-charged [M-16 + H]^+^ and [M + Na]^+^ ions observed at *m*/*z* 809.9 and *m*/*z* 847.4, and double-charged species that were assigned to the sodium ([M + Na + H]^2^^+^, *m*/*z* 421.7) and ammonium adducts ([M + NH_4_ + H]^2^^+^, *m*/*z* 424.2).

**Figure 4:**
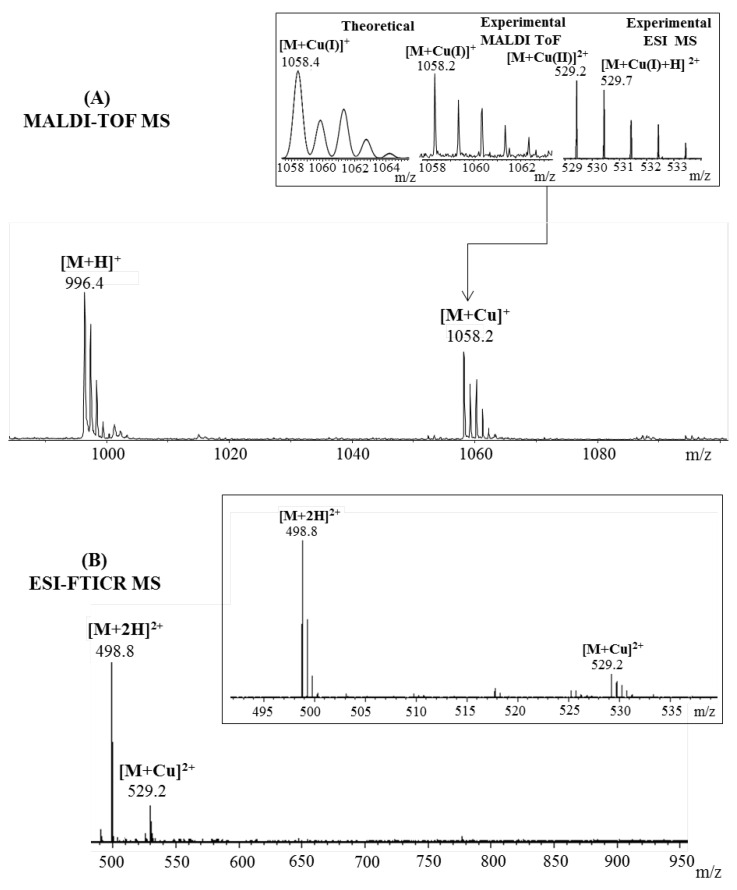
Enlarged region of mass spectra of Aβ(9–16) peptide incubated with Cu^2^^+^ ions acquired by (**A**) MALDI ToF (reflectron mode) with CHCA matrix and (**B**) ESI-FTICR, both in positive mode. Inserts: (**A**) details of theoretical and experimental isotopic patterns of [M + Cu]^+^ ion; (**B**) detail of the double-positive-charged peptide fragments.

The expected copper–peptide complex was also observed in the mass spectra of amyloid Aβ(9–16) peptide fragment (Figure 4). Thus, a strong signal observed at *m*/*z* 1058.2 in the MALDI MS spectrum corresponded to the [M + Cu]^+^. Similar to the previous case, the ionization technique which involves a mechanism of gas-phase charge exchange with matrix molecules favored the reduction of copper (II) ions to copper (I). However, this process did not restrain the metal ions affinity toward Aβ(9–16) peptide. Beside the Cu–Aβ(9–16) complex species, another intense signal visible at *m*/*z* 996.4, generated by the non-complexed peptide, was present in the MS spectrum. Furthermore, no single-charged ions were observed after electrospray peptide’s ionization. Thereby, the only peaks observed at *m*/*z* 498.8, assigned to the protonated peptide [M + 2H]^2^^+^ and *m*/*z* 529.2, corresponding to the Cu–peptide species [M + Cu]^2^^+^, belonged to the double-charged domain. Furthermore, to confirm the presence of a metal–peptide complex that involves copper(II) species, the peak region was zoomed (Figure 4A insert, right spectra) and compared to the theoretical one. Thus, by comparing the isotopic pattern, it was highlighted that the copper ion is found in complexes in both oxidation states.

However, by looking at the intensity of the isotopes, the highest influence seems to come from [M + Cu(II)]^2^^+^ species visible at *m*/*z* 529.2, while the reduced [M + Cu(I) + H]^2^^+^ ions observed at *m*/*z* 529.7 overlap with the isotopic distribution of the precedent complex. These results are in accordance with previous findings [25] where the authors identified a mixture of Cu(I)– and Cu(II)–Aβ(9–16) complexes by electrospray ionization mass spectrometry.

### 2.2. Peptides Affinity toward Metal Ions: Competition Study

**Figure 5:**
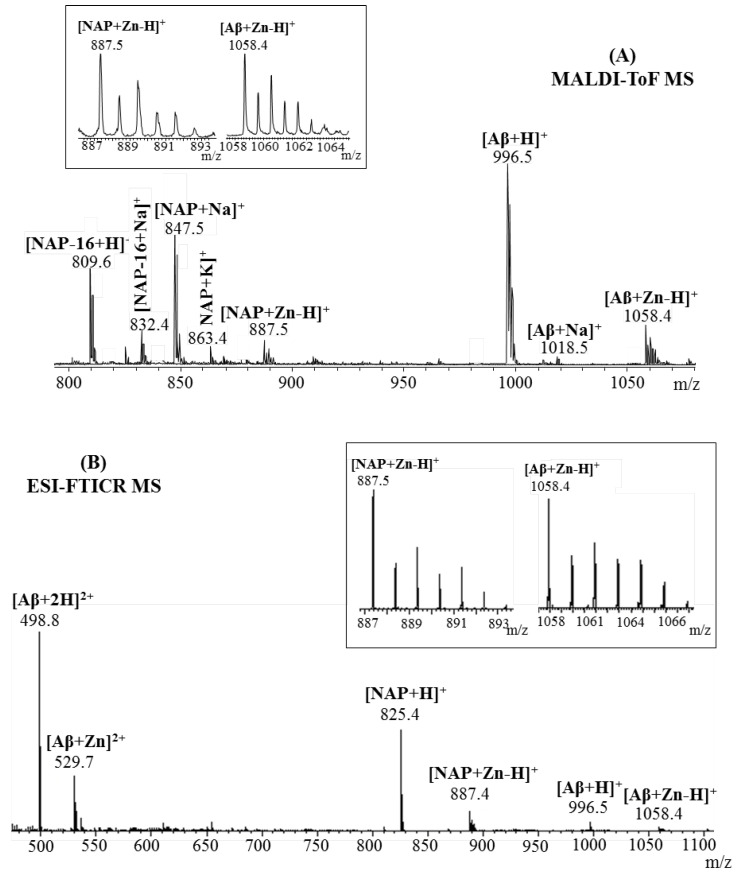
Enlarged region of mass spectra of both NAP and Aβ(9–16) peptide incubated with Zn^2+^ ions acquired by (**A**) MALDI ToF (reflectron mode) with CHCA matrix and (**B**) ESI-FTICR, both in positive mode. Inserts: (**A**) details of theoretical and experimental isotopic patterns of [M + Zn-H]^+^ ion; (**B**) details of the double-positive-charged peptide fragments.

Figure 5 presents the MS spectra recorded after in-solution NAP and Aβ(9–16) peptides incubation with zinc ions. As observed in the (A) spectrum, both peptides formed non-covalent complexes with zinc ions. Thus, in addition to the signals assigned to the protonated [M + H]^+^, deaminated [M-16 + H]^+^ or sodium ion adducts [M + Na]^+^, the peptides generated peaks characteristic of the zinc–peptide complex [M + Zn-H]^+^. This result indicates that there is no relevant interference between the amyloid fragment and neuroprotective NAP peptide and their metal ions binding sites.

Additionally, an ESI-MS experiment was performed in order to reflect, with good accuracy, the solution conditions. The zinc–peptide interaction was easily confirmed by the presence of strong signals at *m*/*z* 1058.4, *m*/*z* 887.4 and *m*/*z* 529.7 corresponding to the single- and double-charged [M + Zn] complex. Therefore, the obtained spectrum (Figure 5B) pointed out that there is no interaction between peptides that would disrupt their affinity for metal ions. Interestingly, Aβ(9–16) peptide was observed predominantly in its doubly protonated forms while the NAP peptide preferred the singly charged ions.

**Figure 6:**
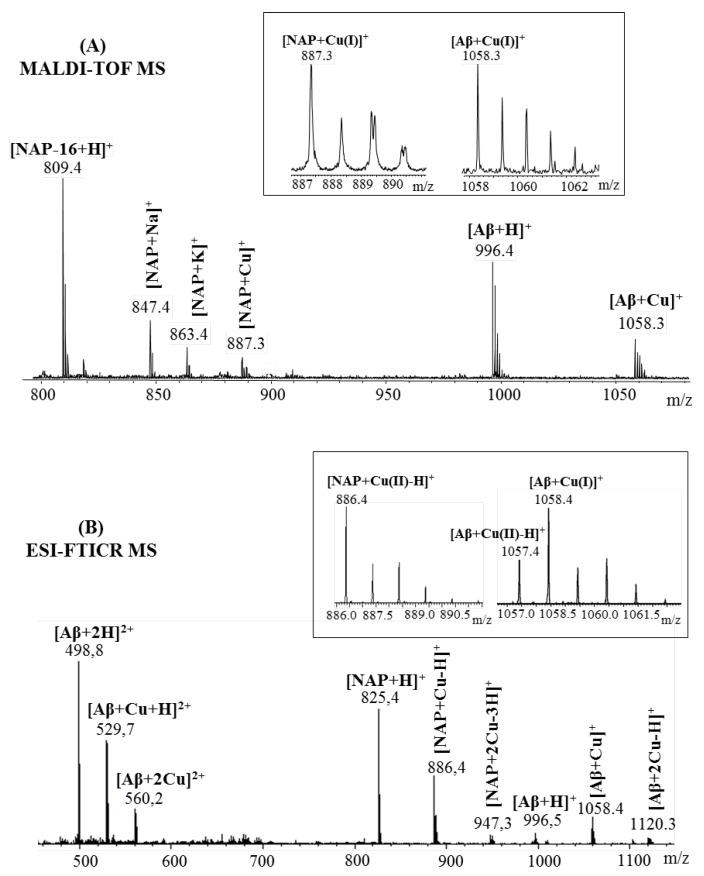
Enlarged region of mass spectra of both NAP and Aβ(9–16) peptide incubated with Cu^2^^+^ ions acquired by (**A**) MALDI ToF (reflectron mode) with CHCA matrix and (**B**) ESI-FTICR, both in positive mode. Inserts: (**A**) details of theoretical and experimental isotopic patterns of [M + Cu-H]^+^ ion; (**B**) details of the double-positive-charged peptide fragments.

In the presence of copper (II) ions, the peptides formed non-covalent complexes with the metal ion. The Cu–peptide complexes were visible in both MALDI and ESI mass spectra (Figure 6), confirming the strong affinity of octapeptides for copper ions. Interestingly, the MALDI ToF/ToF analysis provided evidence of a copper (I)–peptide interaction for both amyloidal and neuroprotective fragments. Further, the analysis of the ESI-MS soft ionization method highlighted the presence of M + Cu(II) ions for NAP peptide while the Aβ9–16 peptide generated signals for both M + Cu(II) and M + Cu(I) species.

In addition, the ability of NAP peptide to bind two copper ions and display signals at *m*/*z* 886.4 ([NAP + Cu-H]^+^) and *m*/*z* 947.3 ([NAP + 2Cu-3H]^+^) confirms the preference of neuroprotective octapeptide for divalent copper ions. Overall, the NAP peptide may play a role in copper homeostasis and/or metabolism since it is able to assimilate the copper redox couple and despite the structural constraints imposed by the proline residues. A similar behavior was also remarked at Aβ(9–16) peptide. However, the reduced Cu+ species was found to be the metal ions predominant in the Cu–Aβ complexes. Furthermore, in the presence of excess Cu, the amyloid fragment showed strong binding of only one Cu+ ion in the MALDI spectrum. In the ESI spectrum, beside the fact that the binding of the second copper ion was substantially weaker, the two peaks assigned to the metal–peptide complex include copper–Aβ complexes with both oxidation states.

### 2.3. Confirmation of Metal Ion Binding by Tandem Mass Spectrometry

In order to determine the metal-binding site for the zinc and copper ions, ESI tandem (MS/MS) experiments were carried out. Tandem mass spectrometry [26] employs two stages of mass analysis in order to examine selectively the fragmentation of a particular ion in a mixture of ions generated in a mass analyzer (in our case, the LIFT cell) [27] by using a collision-neutral gas such as nitrogen. Thus, by analyzing the molecular masses of generated fragments from a selected parent ion, it is easy to identify the specific residues that interact with the metal ions due to their corresponding increase in mass. Thereby, the main metal–peptide signal ions in the mass spectra were selected as parent ions and fragmented in the CID chamber. 

**Figure 7:**
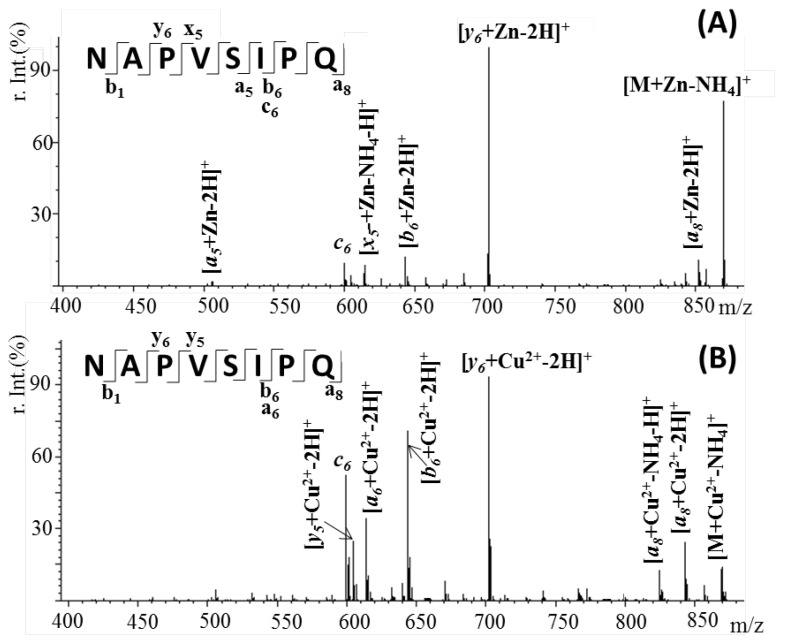
Tandem mass spectrometry (MS/MS) spectrum of the singly charged (**A**) [M + Zn-H]^+^ion at *m*/*z* 887.4 and (**B**) [M + Cu-H]^+^ ion at *m*/*z* 886.4, showing the x, y, z and a, b fragments of the peptide sequence.

Figure 7 presents the tandem MS spectra of [M + Zn-H]^+^ (*m*/*z* 887.4) and [M + Cu-H]^+^ (*m*/*z* 886.4) ions of octapeptide NAP complex that resulted from collision-induced dissociation. The presence of a strong signal assigned to the deaminated Zn–peptide complex [M + Zn-NH_4_]^+^ provides evidence that the binding site is not located within the N-terminal region of the peptide. The most intense fragments observed in both spectra were those assigned to the y6-b6 pair (Table 1). Meanwhile, the smallest fragments identified to bind Zn^2^^+^ were a5 and x5, respectively, suggesting that the metal ion interacts with the serine residue located at position 5 in the ^3^PVS^5^ peptide fragment. A related signal was observed in the case of copper ions where the presence of an y5 Cu-binding fragment confirms the role of Ser as a metal-binding site. Based on these results, it is likely that coordination in both cases involves the side-chains of the P, S and Q residues present in this region, as previously suggested by other methods [28,29].

**Figure 8:**
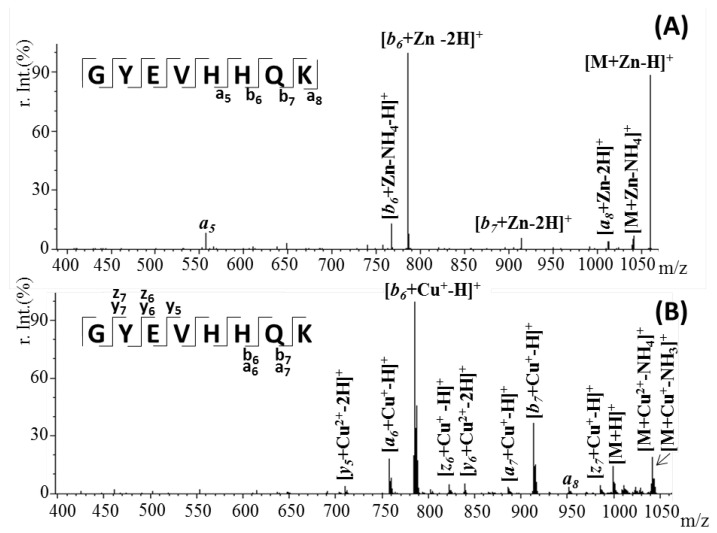
Tandem mass spectrometry (MS/MS) spectrum of the doubly charged (**A**) [M + Zn]^2^^+^ ion at *m*/*z* 529.7 and (**B**) [M + Cu]^2^^+^ ion at *m*/*z* 529.2, showing the y, z and a, b fragments of the peptide sequence.

Similar to the NAP peptide, the metal–Aβ(9–16) doubly charged species preserved the protein–metal interactions, while the amide backbone of the peptide was cleaved. As observed in Figure 8, the most intense signal, registered in both spectra, was assigned to the b6 metal-binding fragment. The presence of a strong signal at *m*/*z* 1058.4, characteristic of the [M + Zn-H]^+^ ions, and absence of other relevant fragments, beside the b6, indicates that zinc forms a strong binding with the histidine-rich fragment and stabilizes the peptide structure. By comparison, in a previous research study, it was observed that in the absence of the metal ion, the peptide fragmentation allows the division of the two histidine residues [30]. Likewise, the copper–peptide complex formed mostly b6 fragments carrying Cu(I) species. This interaction observed in the CID spectrum of [M + Cu]^2+^ ions was most likely mediated through the histidine residues in this region. In addition, besides the fragments holding the reduced copper ions, the MS^2^ spectrum generated signals, characteristic of the y6, y5 and the unfragmented molecular ion, containing the Cu(II) species (Table 2). However, the intensity of those peaks is insignificant compared to the ones attributed to Cu^+^-associated fragments.

### 2.4. Morphological and Topographical Characterization/Atomic Force Microscopy Investigation

Atomic force microscopy (AFM) is a powerful profilometry technique that gives quantitative information about the surface microstructure of thin films. AFM also provides morphological information in terms of roughness and heights of the structures [31]. In addition to characterizing different films, this method has utility in characterizing self-assembled nanostructures of peptides [32,33]. Hane F. et al. previously demonstrated by AFM that Cu^2^^+^ influences amyloid-(1-42) aggregation by increasing peptide–peptide binding forces [34].

**Figure 9:**
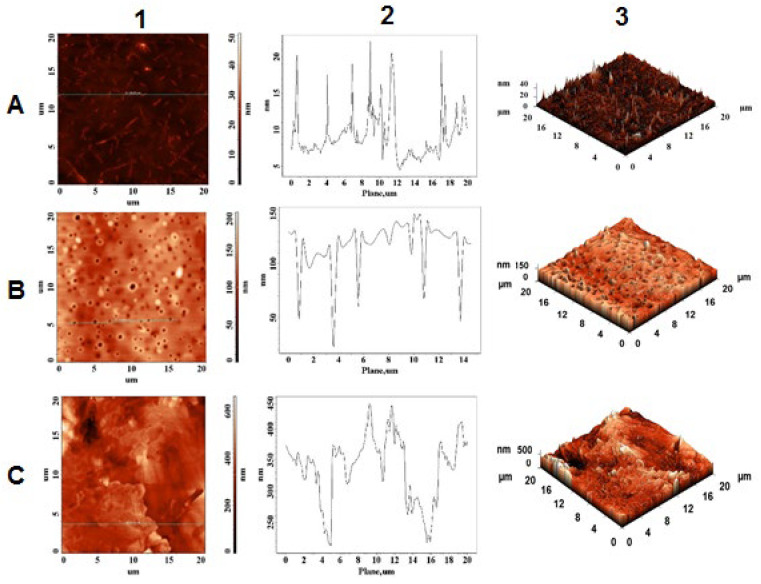
AFM images of NAP peptide (**A**); in the presence of copper ions (**B**) and zinc ions (**C**); (1) height of AFM images; (2) plot height distribution of particles along the profile line; and (3) 3D AFM images.

AFM experiments conducted on fresh solution of peptides formed a thin film on the microscope slide. As observed in Figure 9A, NAP peptide forms, in its native state, homogeneous elongated crystals with a maximum height of 22 nm.

**Figure 10:**
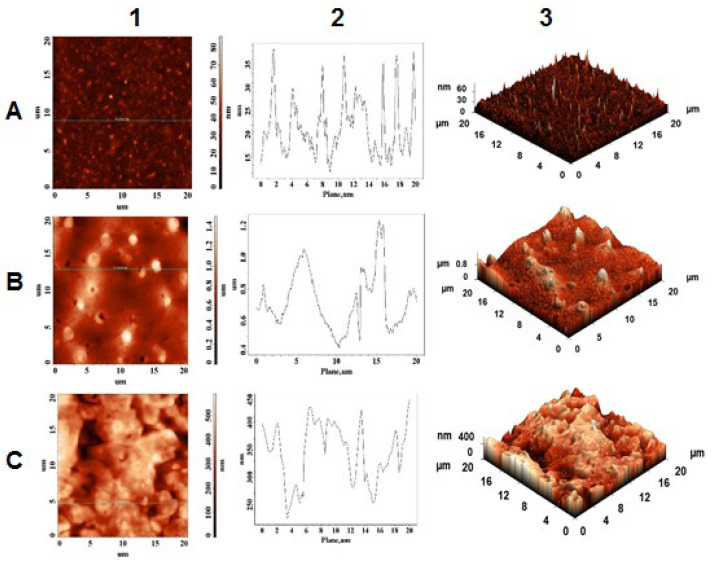
AFM images of Abeta9–16 peptide (**A**); in the presence of copper ions (**B**) and zinc ions (**C**); (1) height of AFM images; (2) plot height distribution of particles along the profile line; and (3) 3D AFM images.

Moreover, Aβ(9–16) peptide (Figure 10A) formed mostly monodisperse spherical crystals characterized by a more convex shape than NAP peptide, having maximum height of 38.5 nm. As anticipated, the presence of metal ions significantly influenced the nanostructures of peptides in aqueous solution. Thus, a strong contrast between peptide crystallization in the absence and presence of peptide ions was recorded by the AFM equipment. For example, AFM images of NAP peptide carried out in the presence of Cu^2^^+^ ions (Figure 9B) showed the formation of a porous layer having a thickness of approximately 130–150 nm whereas the Aβ(9–16) fragment (Figure 10B) displayed, on the 0.5–1.2 μm thick layer, a reduction in the density of native Aβ crystals with amorphous and flattened tendency. The difference in topography may be related to the peptides’ structure and their affinity toward copper ions. Meanwhile, the addition of Zn^2^^+^ ions induced the formation of a solid layer having a maximum height of approximately 450 nm. In addition, according to AFM images, the irregular layer formed in the presence of zinc ions preserves some of the peptide crystals, suggesting a lower interaction with the peptides.

**Figure 11:**
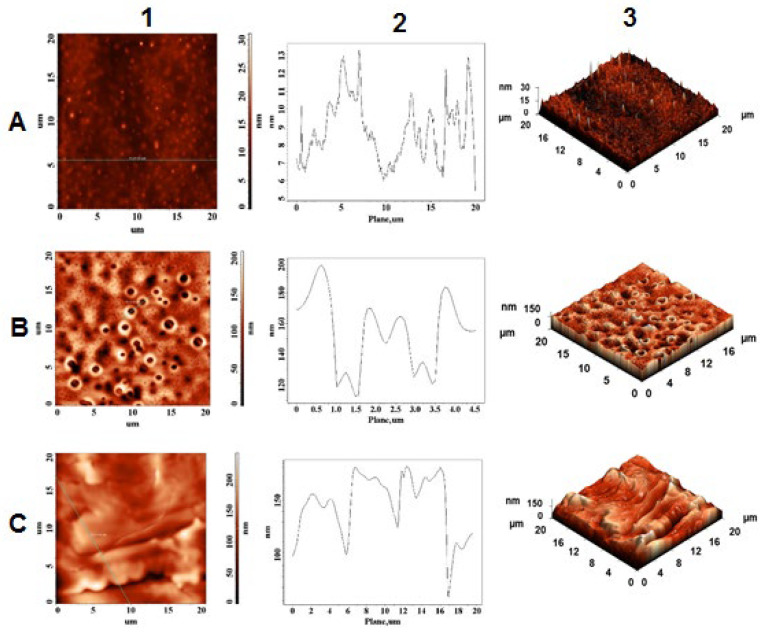
AFM images of NAP and Aβ(9–16) peptide mixture (**A**) in the presence of copper ions (**B**) and zinc ions (**C**); (1) height of AFM images; (2) plot height distribution of particles along the profile line; and (3) 3D AFM images.

The interaction between the two peptides was also investigated. Thus, as observed in Figure 11A, the mixture system formed a slim layer with a maximum height of 13 nm. Compared to pure peptides, shrinkage in crystal height was noticed. Furthermore, spherical crystals of Aβ(9–16) peptide were found to encapsulate NAP peptide, thus blocking amino acids that may interact with metals. As observed in Figure 11B, in the presence of copper(II) ions, the peptide system formed a porous layer characterized by a maximum height of 200 nm and a pore diameter of 0.5 μm. Meanwhile, the intensities of peptides in the presence of Zn^2^^+^ ions were significantly lower than those registered on individual samples, which means that both peptides could chelate zinc ions and alleviate the reduction in intensities of the smooth film.

## 3. Discussion

Alzheimer’s disease has been a priority among research efforts into neurodegenerative diseases, and due to the fact that there is still no treatment, but only methods to alleviate or delay severe symptoms, research in the field continues through different approaches. Lately, there is growing interest in identifying bioactive compounds capable of preventing amyloid fibrils formation associated with neurodegenerative diseases. Among cell-penetrating peptides [35], peptide–drug conjugates hold great promise as an exciting area of research for targeted therapeutic approaches [36]. Various hybrid drugs were designed by combining the therapeutic characteristics of bioactive peptides with small organic molecules for treating different pathologies such as neurodegenerative diseases [37,38], cancer [39,40] and others [41].

In the present study, we examined the interaction between metal ions and two peptides that were previously synthesized [21,42] and are known to be involved in neurodegenerative pathogenesis. NAP exhibits a neuroprotective role and the Aβ(9–16) model peptide fragment represents the N-terminal part of aggregating amyloidogenic peptides, responsible for metal complexation, but not for the formation of neurotoxic fibrils. Both peptides exhibit an affinity to forming complexes with both metal ions (Zn^2^^+^ and Cu^2^^+^) in independent experiments, showing a clear mass spectrometric signature. Both peptides, NAP and Aβ(9–16), formed stable ion complexes with Zn^2^^+^, as shown by MALDI and ESI mass spectrometric measurements, incorporating a single Zn atom into the peptide sequence. In contrast, Cu–peptide interaction studies by the ESI-MS soft ionization method highlighted the presence of M + Cu(II) ions for NAP peptide while the Aβ(9–16) peptide generated signals for both M + Cu(II) and M + Cu(I) species. In addition, the ability of NAP peptide to bind two copper ions confirms the preference of neuroprotective octapeptide for divalent copper ion interactions.

Further, tandem mass spectrometry using the LIFT fragmentation approach was used to specifically identify the metal ion binding site within the two peptides. In the case of the NAP peptide, we observed that the Zn metal ion interacts with the serine residue located in the ^3^PVS^5^ peptide fragment, while Cu interactions need involvement of the next proline residues. Tandem MS of Aβ(9–16)–zinc or copper complexes showed strong binding with the histidine-rich fragment that stabilizes the peptide structure.

Moreover, in-solution incubation of both peptides with zinc ions showed that there is no interaction between peptides that would disrupt their affinity for metal ions, suggesting that NAP and Aβ(9–16) peptide form independent zinc complexes. In the case of concomitant copper–peptides interaction, we observed a strong affinity and preference for divalent copper ions of NAP octapeptides and strong binding of only one Cu+ ion in the case of the amyloidogenic fragment.

Our findings show that the N-terminal Aβ(9–16) fragment can bind metal ions due to its rich histidine center, causing it to stiffen in comparison to its normal in-solution flexibility. The Aβ(9–16)–metal interactions help to promote the aggregating species of the pathophysiological Aβ(1-40/42).

The morphological and topographical characterization of both peptides in the absence and in the presence of zinc and copper ions was investigated by atomic force microscopy. As discussed in Section 2.4, the peptides showed a specific morphological and topographical signature that is reflected in their in-solution interaction with the zinc and copper ions. These in vitro studies show the structural diversity that peptides adopt in simple experimental work environments, suggesting their complexity in in vivo experiments.

We are convinced that natural or synthetic peptides offer enormous growth as future therapeutics in numerous human pathologies, such as development in cell-penetrating peptides, peptide drug conjugates and peptide metal chelators.

## 4. Materials and Methods

### 4.1. Materials

Peptide synthesis was performed using as solid support Fmoc-Gln(Trt)-Wang Resin (0.4–0.8 mmol amino acid/g of resin) purchased from NovaBiochem (Darmstadt, Germany). The amino acids required for synthesizing the desired peptides sequence were protected at N-terminal with Fmoc group (9-fluorenylmethyloxycarbonyl) and were obtained from GL Biochem (Shanghai, China). Other materials used for peptide synthesis were obtained from Merck (Germany). The metal salts were supplied by Sigma Aldrich and used for the preparation of in-solution metal–peptide complexes. The experimental solutions were prepared using deionized water (18.2 MΩ∙cm) produced by a Milli-Q system (Millipore, Bedford, MA, USA). All other reagents were used without further purification.

### 4.2. Peptide Synthesis

The synthesis of the required peptides was carried out manually using classical solid phase Fmoc/tBu solid phase synthesis (SPPS). The NAP octapeptide (NH_2_-NAPVSIPQ-COOH) was prepared starting from an Fmoc-Gln(Trt)-Wang resin, while for the Aβ(9–16) fragment, Fmoc Rink Amide resin (H_2_N-GYEVHHQK-CONH_2_) was used. Summarily, the peptides synthesis was performed in a fritted plastic reactor connected to a vacuum pump to remove washing solutions by successive cycles of Fmoc/tert-butyl deprotection and amino acid addition. All reactions were performed in dimethylformamide (DMF) medium: protecting groups such as Fmoc or tert-butyl were removed with 20% piperidine while activation of the new amino acid was accomplished in the presence of benzotriazol-1-yl-oxytripyrrolidinophosphonium hexafluorophosphate (PyBOP) and N-methylmorpholine (NMM). Final cleavage of the peptides from the resin was performed with a TFA:TIS:H_2_O solution, in a ratio of 95:2.5:2.5 (*v*/*v*/*v*). The precipitation of the peptide was performed cold (−20 °C) in a solution of ethyl ether. Finally, the resulting fractions, eluted with 5% acetic acid solution in ultrapure water, were lyophilized and stored at −20 °C until further use.

### 4.3. Mass Spectrometry and Peptide Complex Formation

For MALDI-ToF experiments, 4 mM stock peptide solutions, prepared in deionized water at a pH of 7.4 adjusted by drop-wise addition of aqueous NaOH, were incubated in the presence of metal salts (CuSO_4_, ZnSO_4_) at a 1:10 peptide to metal molar ratio. The resulting mixture was gently mixed for 20 h at 24 °C and 350 rpm in a thermo-shaker (Thermomixer Compact Eppendorf AG 22331, Hamburg, Germany). MALDI-ToF MS was performed on a Bruker Ultraflex MALDI ToF/ToF mass spectrometer equipped with a pulsed nitrogen UV laser. The instrument was run in positive ionization mode and measurements were performed in the reflectron mode using α-cyano-4-hydroxycinnamic acid (HCCA) as matrix. The samples were loaded onto a 384-spot target plate of the MALDI-ToF instrument using the dried-droplet method by spotting 0.5 μL of matrix solution and 0.5 μL of sample solution, which were mixed directly on the target and allowed to dry in the ambient air. Calculations conducted with the ChemCalc online program were employed for theoretical monoisotopic mass determination. The spectra were recorded using the following parameters: 20 kV acceleration voltage, 40% grid voltage, 140 ns delay, low mass gate of 500 Da and an acquisition mass range of 600–3500 Da. In the final mass spectrum, 300 shots per acquisition were accumulated. External calibration was carried out using the monoisotopic masses of singly protonated ion signals of Bradykinin (1–7) (*m*/*z* 757.4), Angiotensin II (*m*/*z* 1046.5), Angiotensin I (*m*/*z* 1296.7), Substance P (*m*/*z* 1347.7), Bombesin (*m*/*z* 1619.8), Renin Substrate (*m*/*z* 1758.9), ACTH clip (1–17) (*m*/*z* 2093.1), ACTH clip (18–39) (*m*/*z* 2465.2) and Somatostatin (*m*/*z* 3147.5). The obtained spectra were processed using Bruker Flex Analysis 3.4 software.

ESI experiments were performed using sample solutions containing 12.5 μM of peptide and 125 μM metal salt. To preserve native peptide–metal interactions, samples were prepared in Milli-Q H_2_O containing 25 mM ammonium acetate (pH 7.4). The ESI MS measurements were performed using a high-resolution mass spectrometer with Fourier transform and ion cyclotronic resonance FT-ICR MS (7T Thermo Finnigan LTQ FT, Bremen, Germany) coupled with an Agilent 1100 nano-HPLC system. The samples were initially diluted in 50% methanol solution and directly injected using a Hamilton syringe into the interface using a Harvard 11 PLUS pump, while the flow was set at 10 μL/min. The ions, obtained using the nano-ESI ionization source in positive ion mode, were accumulated externally in a hexapole collision cell before being transferred to the LTQ cell and subjected to a CID. The parent ions were isolated in a window with a width of 5–10 *m*/*z* with the center mass upshifted by 0.5–1 from the parent. CID fragmentation was conducted with normalized collision energy in the range of 15 to 25, and fragment spectra were acquired in the FTICR cell with a resolution of 25–50 k in the mass range 240–1100 with 1 micro scan and 500 ms max injection time at an AGC setting of 2 × 10^5^. The spray voltage used for positive ionization was ∼2800 V. The mass spectra were obtained within the mass range *m*/*z* 200–2000 with a resolution of 50,000 at AGC 1 × 10^6^ and with an injection time of maximum 500 THX. The standard FT calibration mixture was used to calibrate the external mass. Fragmentation of precursor ions was accomplished by LIFT cell in MALDI–ToF/ToF mass analyzer [27]. The obtained MALDI spectra were processed using the FlexAnalysis program while for ESI spectra we used mMass software. Moreover, the correct peptide mass, theoretical fragmentation ions, hydrophobicity plots and peptide-charged ions were theoretically calculated using the GPMAW program.

### 4.4. Atomic Force Microscopy (AFM)

AFM experiments were carried out at a 1:10 peptide: metal molar ratio. In total, 5 μL of each sample was deposited onto a 1 × 1 cm cleaved mica surface and allowed to dry for 24 h at RT covered by a Petri dish to avoid dust. The surface images were obtained with an NTEGRA scanning probe microscope (NT-MDT Spectrum Instruments, Moscow, Russia), in AFM configuration. Rectangular silicon cantilevers NSG10 (NT-MDT, Moscow, Russia) with tips of high aspect ratio (sharpened pyramidal tip, angle of nearly 20°, tip curvature radius of 10 nm and height of 14–16 µm) were used in order to minimize convolution effects. All images were acquired in air, at RT, in tapping mode, with the velocity of 6 mm/s. For image acquisition, the Nova v.19891 for Solver software was used. All AFM images were obtained at a resolution of 256 × 256 pixels on a scale of 20 µm × 20 µm.

## Figures and Tables

**Table 1:** Collision-induced dissociation of [M + Zn-H]^+^ and [M + Cu-H]^+^ ions.

Fragmented Ion	Sequence	Predicted (*m*/*z*)	Observed (*m*/*z*)
**[M+ Zn-H]^+^**	[M + Zn-NH_4_]^+^	871.1988	871.2012
	[a_8_ + Zn-2H]^+^	842.4358	842.4398
	[y_6_ + Zn-2H]^+^	703.3127	703.3145
	[b_6_ + Zn-2H]^+^	645.2395	645.2457
	[x_5_ + Zn-NH_4_-H]^+^	615.4617	615.4673
	c_6_	599.3511	599.3515
	[a_5_ + Zn-2H]^+^	504.3096	504.3138
**[M** **+ Cu-H]** **^+^**	[M + Cu^2^^+^-NH_4_]^+^	869.3648	869.3651
	[a_8_ + Cu^2^^+^-2H]^+^	840.4818	840.4827
	[a_8_ + Cu^2+^-NH_4_-H]^+^	823.4428	823.443
	[y_6_ + Cu^2^^+^-2H]^+^	701.3787	701.3782
	[b_6_ + Cu^2^^+^-2H]^+^	643.2024	643.1998
	[a_6_ + Cu^2^^+^-2H]^+^	615.1836	615.1843
	[y_5_ + Cu^2^^+^-2H]^+^	604.1872	604.1902
	c_6_	599.3511	599.3524

**Table 2:** Collision-induced dissociation of [M + Zn]2+ and [M + Cu]2+ ions.

Fragmented Ion	Sequence	Predicted (*m*/*z*)	Observed (*m*/*z*)
**[M + Zn-H]^+^**	[M + Zn-H]^+^	1060.3988	1060.4022
	[M + Zn-NH_4_]^+^	1043.4514	1043.4437
	[a_8_ + Zn-2H]^+^	1014.2408	1014.2487
	[b_7_ + Zn-2H]^+^	914.3472	914.3408
	[b_6_ + Zn-2H]^+^	786.2281	786.2245
	[b_6_ + Zn-NH_4_-H]^+^	769.2502	769.2429
	a_5_	558.2040	558.1998
**[M + Cu-H]^+^**	[M + Cu^+^-NH_3_]^+^	1043.3867	1043.3816
	[M + Cu^2+^-NH_4_]^+^	1042.3859	1042.3842
	[z_7_ + Cu^+^-H]^+^	985.4288	985.4268
	a_8_	951.4534	951.4452
	[b_7_ + Cu^+^-H]^+^	913.3502	913.3427
	[a_7_ + Cu^+^-H]^+^	885.2957	885.2904
	[y_6_ + Cu^2+^-2H]^+^	838.3398	838.3342
	[z_6_ + Cu^+^-H]^+^	822.3448	822.3403
	[b_6_ + Cu^+^-H]^+^	785.2418	785.2442
	[a_6_ + Cu^+^-H]^+^	757.2336	757.2369
	[y_5_ + Cu^2+^-2H]^+^	709.3309	709.3378

## Data Availability

Data is contained within the article.
